# The *locus coeruleus* Is Directly Implicated in L-DOPA-Induced Dyskinesia in Parkinsonian Rats: An Electrophysiological and Behavioural Study

**DOI:** 10.1371/journal.pone.0024679

**Published:** 2011-09-09

**Authors:** Cristina Miguelez, Asier Aristieta, Maria Angela Cenci, Luisa Ugedo

**Affiliations:** 1 Department of Pharmacology, Faculty of Medicine and Dentistry, University of the Basque Country, Vizcaya, Spain; 2 Basal Ganglia Pathophysiology Unit, Department of Experimental Medical Science, Lund University, Lund, Sweden; The Mental Health Research Institute of Victoria, Australia

## Abstract

Despite being the most effective treatment for Parkinson’s disease, L-DOPA causes a development of dyskinetic movements in the majority of treated patients. L-DOPA-induced dyskinesia is attributed to a dysregulated dopamine transmission within the basal ganglia, but serotonergic and noradrenergic systems are believed to play an important modulatory role. In this study, we have addressed the role of the *locus coeruleus* nucleus (LC) in a rat model of L-DOPA-induced dyskinesia. Single-unit extracellular recordings in vivo and behavioural and immunohistochemical approaches were applied in rats rendered dyskinetic by the destruction of the nigrostriatal dopamine neurons followed by chronic treatment with L-DOPA. The results showed that L-DOPA treatment reversed the change induced by 6-hydroxydopamine lesions on LC neuronal activity. The severity of the abnormal involuntary movements induced by L-DOPA correlated with the basal firing parameters of LC neuronal activity. Systemic administration of the LC-selective noradrenergic neurotoxin N-(2-chloroethyl)-N-ethyl-2-bromobenzylamine did not modify axial, limb, and orolingual dyskinesia, whereas chemical destruction of the LC with ibotenic acid significantly increased the abnormal involuntary movement scores. These results are the first to demonstrate altered LC neuronal activity in 6-OHDA lesioned rats treated with L-DOPA, and indicate that an intact noradrenergic system may limit the severity of this movement disorder.

## Introduction

Parkinson’s disease (PD) is a progressive neurodegenerative process that causes the most common movement disorder of basal ganglia origin [1]. The motor impairment in PD arises from the selective loss of dopaminergic neurons in the *substantia nigra pars compacta* and the subsequent reduction of dopamine levels in the striatum [2]. Currently, pharmacological dopamine replacement with L-DOPA is the gold standard treatment for PD. However, long-term administration of L-DOPA induces abnormal involuntary movements known as L-DOPA-induced dyskinesias (LID). These motor complications are discomforting and potentially disabling, and affect up to 40% of PD patients within 5 years of treatment [3]. There is vast consensus that LID results from dysregulated dopamine neurotransmission depending on both presynaptic alterations and post-synaptic dopamine receptor supersensitivity (reviewed in [4], [5], [6], [7]). However, there is also evidence implicating the noradrenergic system in LID: (1) L-DOPA may be transformed into noradrenaline [8], and radioligand binding data demonstrate that dopamine produced from L-DOPA and some L-DOPA metabolites bind with high affinity not only D1 and D2 dopamine receptors, but also to α_2A_ and α_2C_-adrenoceptors [9]; (2) post-mortem studies have revealed a substantial loss of noradrenergic neurons [10], [11], a decrease in noradrenaline levels in the brain [12], [13] and reduced levels of the noradrenaline transporter in several noradrenergic areas in the brains of PD patients [14]; (3) studies using α_2_-adrenoceptor antagonists, such as idazoxan, yohimbine and fipamezole, show a significant reduction in dyskinesia in 6-hydroxydopamine (6-OHDA)-lesioned rats [15], 1-methyl-4-phenyl-1,2,3,6-tetrahydropyridine (MPTP)-lesioned primates [16], [17], [18], [19] and PD patients [20]. Clonidine, an α_2_-adrenoceptor agonist, also reduces LID in rodent models and PD patients [15], [20]. In addition, α_1_-adrenoceptors contribute to L-DOPA-induced hyperactivity in MPTP-lesioned macaques [21].

The largest population of central noradrenergic neurons is located in the *locus coeruleus* (LC), which undergo degeneration in PD [22]. Under control conditions, a substantial amount of dopamine is present in the LC [23], [24], [25], where it is used as a precursor of noradrenaline or as a neurotransmitter itself. Dopamine inhibits LC neuronal electrical activity [26], [27] but also stimulates LC cells by acting on α_1_-adrenoceptors [25]. Recently, we have shown that nigrostriatal degeneration decreases LC noradrenergic neuronal basal firing rate [28]. Studies examining the impact of LC lesions on the severity of L-DOPA-induced dyskinesia have produced conflicting results. In one study [29], rats with a combined lesion of noradrenergic and dopaminergic systems expressed more severe LID than rats with conventional dopaminergic lesions [30]. In other studies, combined lesions of the dopaminergic and noradrenergic pathways increased the motor response to L-DOPA [31] but did not modify motor response alterations induced by chronic L-DOPA treatment, measured as a shortening in the duration of contralateral rotation [32].

In this study, we have tested the hypothesis that LC neuronal activity plays an important modulatory role in LID. The study was performed in 6-OHDA-lesioned rats that were rendered dyskinetic with a course of daily L-DOPA treatment. In separate experiments, we examined the effects of LC damage on the severity of LID and we recorded single unit extracellular activities from LC neurons. Our data demonstrate that chemical local lesions of the LC increase L-DOPA-induced abnormal involuntary movement scores, and that these scores are strongly correlated with several parameters of LC noradrenergic neuronal activity. Taken together, these results provide both electrophysiological and behavioural support to an involvement of the LC in the development of LID.

## Materials and Methods

### Subjects

Female Sprague-Dawley rats (220–225 g) were housed under a 12:12 h light:dark cycle with food and water provided *ad libitum*. Every effort was made to minimise animal suffering and to use the minimum number of animals possible. Experimental protocols were reviewed and approved by the Local Ethical Committee for Animal Research at the University of the Basque Country and Lund University. All of the experiments were performed in compliance with the European Community Council Directive on “The Protection of Animals Used for Experimental and Other Scientific Purposes” (86/609/EEC) and with Spanish Law (RD 1201/2005) for the care and use of laboratory animals.

### Experimental design and groups

**Figure 1:**
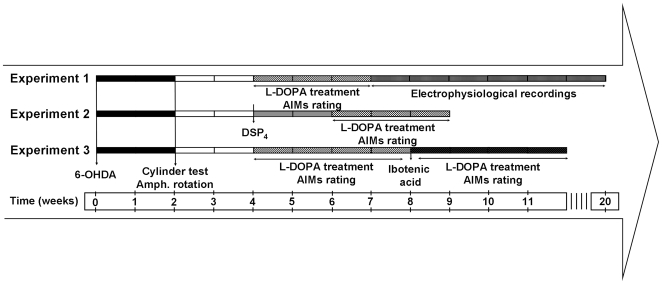
Experimental design. ***Experiment 1***: At the beginning of the study, animals were lesioned with 6-OHDA injected into the right MFB. Two weeks later, the severity of the lesion was screened by forelimb use on a cylinder and amphetamine-induced rotations. To develop stable AIMs, rats were injected daily with 6 mg/kg L-DOPA (plus 12 mg/kg benserazide) or saline for 21 days, and AIMs were rated 2-3 days per week. At the end of the chronic treatment, L-DOPA was administered twice per week, and electrophysiological experiments were performed. ***Experiment 2***: Animals were lesioned with 6-OHDA injected into the right MFB, and the severity of the lesion was screened in the 4^th^ week. DSP-4 (50 mg/kg, i.p.) was administered to some groups to induce an additional noradrenergic lesion. Between the 6^th^ and 9^th^ weeks, animals were treated daily with L-DOPA (6 mg/kg plus 12 mg/kg benserazide, s.c.) or saline, and AIMs were monitored 2-3 times per week. ***Experiment 3***: Animals were lesioned, screened and AIMs were induced and evaluated. The severity of the AIMs was maintained by administering L-DOPA twice per week. On day 30 after the 6-OHDA lesion, dyskinetic animals were injected with ibotenic acid or vehicle into the right LC. Five days after noradrenergic damage, AIMs were evaluated regularly for four weeks. At the end of each study, all animals were transcardially perfused, and the brains were extracted, cut and stored for subsequent histological verification or immunohistochemical procedures.

As it is illustrated in Figure 1, this study was composed of three different experiments.

#### Experiment 1

6-OHDA-lesioned and control animals were treated chronically with either L-DOPA or saline. In all groups, the electrophysiological characteristics of LC neurons were analysed 24 h after the last dose of the corresponding drug. The groups included in this experiment are referred to as sham saline (n = 8), sham L-DOPA (n = 7), 6-OHDA saline (n = 9) and 6-OHDA L-DOPA (dyskinetic or non-dyskinetic, n = 8 and 3, respectively).

#### Experiment 2

DSP-4 or saline was injected in 6-OHDA-lesioned and control animals. Two weeks later, animals were chronically treated with L-DOPA or saline, and abnormal involuntary movements (AIMs) were monitored. The different groups of animals included in this study were 6-OHDA (n = 10), 6-OHDA+DSP-4 (n = 10), DSP-4 (n = 6) or sham animals (n = 5).

#### Experiment 3

In eight dyskinetic animals, ibotenic acid was injected into the right LC to destroy the noradrenergic nucleus on day 31st after the beginning of L-DOPA chronic treatment. Five days after LC lesion, AIMs were evaluated for another 32 days in 10 additional testing sessions. The severity of LID before and after the LC lesion was compared. In five additional dyskinetic animals, a vehicle injection (Dulbecco’s buffered saline solution) was performed in the right LC on day 31^st^ and the same protocol as the one used for the LC-lesion group was used. This sham group was introduced in order to exclude an effect of the surgery on the expression of dyskinesia.

### Neurotoxic lesions

#### 6-Hydroxydopamine lesion

At the beginning of the study, 6-hydroxydopamine (6-OHDA-HCl; Sigma-Aldrich) was injected into the right median forebrain bundle (MFB) according to our established procedures [33]. 6-OHDA is a neurotoxin commonly used to lesion dopaminergic pathways and generate experimental models for Parkinson disease. Briefly, rats were anesthetized with isofluorane and mounted on a Kopf stereotaxic frame, two injections of 7.5 and 6 µg 6-OHDA (free-base) were then performed at two coordinates at a rate of 0.5 µl/min. In order to project noradrenergic fibres, thirty minutes before lesioning, desipramine (25 mg/kg, i.p.) was administered.

#### DSP4 administration

Saline or N-(2-chloroethyl)-N-ethyl-2-bromobenzylamine (DSP-4) (50 mg/kg, i.p.) was administered to 6-OHDA-lesioned or sham animals. In both groups, citalopram (20 mg/kg, i.p.) was also administered. DSP-4 exerts neurotoxic actions on noradrenergic neurons and selectively damages noradrenergic projections originating from the LC. DSP-4 solution was made fresh and placed in an opaque, tightly sealed container due to its instability and light sensitivity as described in [34].

#### LC lesioning with ibotenic acid

Animals were anaesthetised with isofluorane and mounted in a stereotaxic frame. The head was oriented at 15° to the horizontal plane (nose down), and 1 µl of ibotenic acid (2 µg/µl) or vehicle was injected into the right LC, AP: -3.7 mm, ML: +1.1 mm, DV: -5.5 relative to lambda [35]. The toxin was infused at a rate of 0.5 µl per min, and the needle was left in place for another 2 minutes before being slowly retracted.

### Behavioural Tests

#### Amphetamine-induced rotation

Two weeks after the lesion with 6-OHDA, animals were injected with 2.5 mg/kg D-amphetamine to screen for the severity of the dopaminergic lesion. Ipsilateral rotations were induced and recorded for 90 min. Only rats rotating more than five full turns per minute, corresponding to a >90% striatal DA depletion [36], [37], were included in the study. Sham animals also received the same dose of D-amphetamine.

#### Cylinder test

Forelimb use asymmetry was measured using the cylinder test, as described previously [38]. Rats were placed individually in a 20-cm diameter glass cylinder and allowed to move freely. Each animal was left in place until it performed at least 20 supporting paw placements on the walls of the cylinder. To obtain an index of forelimb use asymmetry, the following formula was used: [contralateral paw placements/(ipsilateral + contralateral paw placements)] ×100. In each study, the cut-off asymmetry score indicative of severe dopaminergic damage was obtained by subtracting 3 standard deviations from the mean value measured in intact rats (corresponded to a 99.73% confidence limit).

#### Abnormal involuntary movement rating

AIMs were induced in 6-OHDA-lesioned rats by chronic daily injections of L-DOPA (6 mg/kg, s.c., in combination with the peripheral decarboxylase inhibitor benserazide, 12 mg/kg, s.c.) over 3 weeks. The development of L-DOPA-induced AIMs was monitored and scored according to a rat dyskinesia scale described extensively in [39]. When a plateau was reached, the daily L-DOPA treatment was changed to a maintenance dose of twice-weekly administration [40], [41]. On the testing days, rats were placed individually in transparent empty plastic cages for at least 10 minutes prior to drug administration. Following the L-DOPA injection, each rat was observed for one minute every 20^th^ minute for 3 hours. Three subtypes of dyskinetic movements (axial, limb and orolingual AIMs) and asymmetric locomotive behaviour (locomotive AIMs) were rated on the basic severity scale first described in [33] where each item is rated on a scale from 0 to 4 based on the proportion of observation time during which it is present. In addition, the amplitude of axial and limb AIMs was rated on a scale from 0 to 4 as first described in [37]. Axial, limb, and orolingual AIMs were analysed separately from locomotive AIMs using the following parameters: *Sum AIMs*, sum of axial, limb and orolingual AIM scores (basic scale) for an entire testing session; *Global AIMs*: for each AIM subtype, the basic severity score was multiplied with the amplitude score on each monitoring period, and all these products were summed for each testing session [42], [43]. The *Time-course of AIMs* after an L-DOPA injection was expressed by the “area under the curve” (AUC) of the Sum AIMs parameter. L-DOPA-treated rats were classified as “dyskinetic” or “non-dyskinetic” prior to the electrophysiological experiments. The “non-dyskinetic” group included animals that either developed mild or no AIMs (basic severity grade 0–1 on each AIM subtype), whereas rats classified as “dyskinetic” showed moderate to severe AIMs (basic severity grade of ≥2 on at least two of the three AIM subtypes) [42].

### Electrophysiological procedures

**Figure 2:**
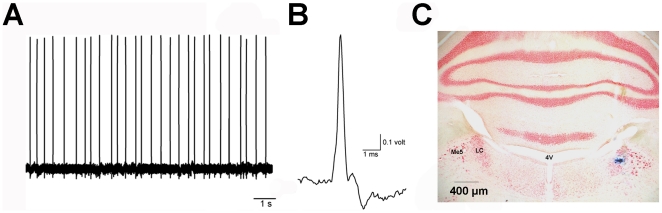
Electrophysiological and histological verification of LC neuron recordings. Example of raw trace of recorded action potentials (A). Scale-up of single spike from LC neuron recorded *in vivo* (B). Pictures were taken from *Spike2* recordings. Histological verification of the recording site in the LC (C). Abbrev: LC, locus coeruleus; 4V, 4th ventricle; Me5, mesencephalic trigeminal nucleus.

Single-unit extracellular recordings of LC neurons were performed as detailed in [44]. Animals were anaesthetised with chloral hydrate (400 mg/kg, i.p.). The rat was placed in a stereotaxic frame, and the body temperature was maintained at ∼37°C for the entire experiment. The head was oriented at 15° to the horizontal plane (nose down), and the recording electrode (filled with a 2% solution of Pontamine Sky Blue in 0.5% sodium acetate) was lowered into the right LC (relative to lambda: AP: -3.7 mm, ML: +1.1 mm, DV: -5.5 to -6.5 mm). LC neurons were identified by standard criteria that included the following characteristics: 1) spontaneous activity displaying a regular rhythm and a firing rate between 0.5–5 Hz (Figure 2A); 2) characteristic spikes with a long-lasting, positive-negative waveform (Figure 2B); and 3) a biphasic excitation-inhibition response to pressure applied to the contralateral hind paw (paw pinch). Burst-firing onset and offset was defined as the concurrence of two spikes with an interspike interval between 0.08 and 0.16 s, respectively [45], [46], [47].

Firing patters were analysed offline using the computer software Spike2, and the following parameters were calculated: firing rate, coefficient of variation (percentage ratio of standard deviation to the mean interval value of an interspike time-interval histogram), the percentage of spikes in burst and the percentage of cells exhibiting burst firing. The number of spontaneously active noradrenergic neurons was determined in 10 stereotaxic electrode tracks first 100 µm concentrically around the first track (9 tracks) and then 200 µm rostral to the initial track (1 track). When one track was not passing through the LC, an additional track rostral to the initial one was performed. The pattern was constant for all animals. Posterior verification of the recording site confirmed that all the recorded neurons belonged to the LC (Figure 2C).

At the end of each experiment, a Pontamine Sky Blue mark was deposited at the recording site by passing a 5 µA cathodic current for 10 minutes through the recording electrode. Animals were deeply anaesthetised and transcardially perfused with 4% ice-cold buffered paraformaldehyde prepared in 0.1 M phosphate buffer. Brains were removed and transferred to a 25% sucrose solution until they sank. The brains were cut in coronal 40-µm sections using a freezing microtome (HM 430, Microm), and slices were conserved in cryoprotective solutions at −20°C until further processing.

### Histological analysis

#### Tyrosine hydroxylase and Dopamine-β-hydroxylase immunohistochemistry

Tyrosine hydroxylase (TH)-immunostaining was used to examine the degree of dopamine denervation in the striatum. The precision of this method to quantify the degree of the lesion is confirmed by the linear relationship between the number of immunoreactive neurons in the substantia nigra pars compacta and the optical density in the striatum [48]. Dopamine-*β*-hydroxylase (DBH)-immunostaining was utilised to evaluate the depletion of the LC noradrenergic projections to the prefrontal cortex as an index of the efficacy of DSP-4 treatment.

For TH-immunostaining, after inactivation of endogenous peroxidases (3% H_2_O_2_ and 10% methanol in potassium-phosphate buffered saline (KPBS) for 30 min), sections were pre-incubated for 1 h in 5% normal goat serum +0.25% Triton X-100 in KPBS. The primary antibody used for the detection of TH (rabbit anti-TH, Pel-Freez Biologicals, P40101-O) was diluted 1∶1000, and sections were incubated for 36 h at 4°C. Subsequently, the sections were treated with a biotinylated goat antibody against rabbit IgG (BA 1000, Vector Laboratories, Burlingame, CA, USA) diluted 1∶200, and sections were incubated for 2 h. Both immunoreagents were diluted in 5% or 2.5% normal goat serum (for the primary and secondary antibodies, respectively) +0.5% Triton X-100. Thereafter, sections were incubated with an avidin–biotin–peroxidase complex (ABC kit, PK-6100, Vector Laboratories, 1 h). Finally, the peroxidase reaction was developed with 3,3′-diaminobenzidine and 0.03 % H_2_O_2_. Sections were rinsed, mounted onto gelatin-coated slides, dehydrated, cleared with xylene and coverslipped.

For DBH-immunostaining, a similar protocol was followed. In this case, a mouse anti-DBH monoclonal antibody 1∶1000 (Chemicon International, Temecula, CA 92590 USA) and a biotinylated horse anti-mouse antibody 1∶200 (BA 2001, Vector Laboratories, Burlingame, CA, USA; diluted in normal horse serum) were used.

#### Thionine and neutral red staining

To verify the lesion with ibotenic acid, thionine staining on brain sections containing the LC was performed. Slices were rinsed once, mounted on glass slides and processed as follows: distilled water (2×5 min), 70% ethanol (10 min), 96% ethanol (2 min), 96% ethanol/10% paraformaldehyde (4/1, 5 min), 96% ethanol (2 min), chloroform/ethyl ether/96% alcohol (8/1/1, 10 min), 96% ethanol (2 min), 100% ethanol (2×2 min), xylene (5 min) and 100% ethanol (2×2 min), 96%, ethanol (2 min), 96% ethanol (10 min), 70% ethanol (5 min), 50% ethanol (5 min), thionine (1 g/100 ml, 20–45 min), distilled water (1 min), distilled water/glacial acetic acid, (1000/3, 1 min and 30 sec), 70% alcohol/glacial acetic acid (1000/3, 1 min and 30 sec), 96% ethanol (2 min), 100% ethanol (2 min), xylene (2×8 min)]. Immediately after the staining the glasses were coverslipped.

For the location of the recording site (Figure 2B), brain sections containing the LC were rinsed 3×10 min before being mounted on gelatinised glass slides and processed as follows: 1% neutral red (10 min), distilled water (2 rinses), 70% ethanol (20 sec), 95% ethanol (20 sec), 100% ethanol (30 sec) and xylene (2 min). Afterwards, glasses were coverslipped and examined microscopically. Only cells recorded within the LC were included in this study.

#### Optical density or cell counting

For TH- and DBH-immunostaining, three sections containing striatum or prefrontal cortex, respectively, were first captured using a digital camera and mean optical density was visualized using image analysis NIH-produced software, ImageJ (http://rsb.info.nih.gov/nih.image/default.html). Images from control and treated groups were processed under the same light and contrast conditions and identical camera setting were used to capture them. For TH-immunostaining, the whole striatum was delineated and its optical density was expressed as a percentage of that on the contralateral intact side after background subtraction (background was measured in the cortex on the side ipsilateral to the lesion). Densitometric analysis of DBH-immunoreactivity was performed only in the prefrontal cortex (bregma +2.70 mm) because the signal from the striatum was very close to background levels. Two sample areas per section were analyzed. The DBH optical density levels were expressed as a percentage of the values from intact controls.

For verifying LC lesion after ibotenic acid injection, morphological and stereological approaches were used. First, as detailed in [48], [49], [50], neuronal loss and glial reactions were evaluated using a light microscope (Nikon Optiphot, x10 objective). Second, stereological cell counting was performed in 5 to 10 regularly spaced sections along the LC by the optical dissector method using the Mercator image analysis system (Explora Nova, France) as described in [28].

### Drugs

The drugs used in this study were L-DOPA (L-3,4-dihydroxyphenylalanine methyl ester hydrochloride), benserazide-HCl, chloral hydrate, desipramine hydrochloride, amphetamine sulphate, ibotenic acid (α-amino-3-hydroxy-5-isoxazoleacetic acid) and DSP-4 (N-(2-Chloroethyl)-N-ethyl-2- bromobenzylamine hydrochloride) (Sigma-Aldrich Co, St Louis, USA). Chloral hydrate, desipramine, L-DOPA, benserazide, DSP-4 and amphetamine were prepared in 0.9% saline; 6-OHDA was prepared in distilled water containing 0.2 mg/ml ascorbate. Ibotenic acid was dissolved in Dulbecco’s buffered saline solution containing (in mM): NaCl 136.9, KCl 2.7, NaH_2_PO_4_ 8.1, KH_2_PO_4_ 1.5, MgCl_2_ 0.5 and CaCl_2_ 0.9 (pH 7.4). Except for chloral hydrate, drugs were prepared on the day of the experiment.

### Statistical analysis of data

Experimental data were analysed using the computer program GraphPad Prism (v. 5.01, GraphPad Software, Inc). The electrophysiological data were analysed by one-way analysis of variance (ANOVA) or Fisher’s exact test for a comparison of the number of cells with burst-firing activity. Comparisons of AIMs scores were carried out using one- or two-way ANOVA. The AUC of AIMs after a single dose of L-DOPA was analysed using a one-way ANOVA comparing several groups. When the evolution of AIMs after LC lesion with ibotenic acid was evaluated, repeated measures two-way ANOVA was utilised. All significant ANOVAs were followed by post-hoc comparisons using the Bonferronís or Dunnett’s test. Linear regression analyses were used to assess correlations between electrophysiological and behavioural data. The level of statistical significance was set at *p*<0.05. Data are presented as group means ± standard error of the mean (S.E.M.).

## Results

### Development of L-DOPA-induced abnormal involuntary movements

**Figure 3:**
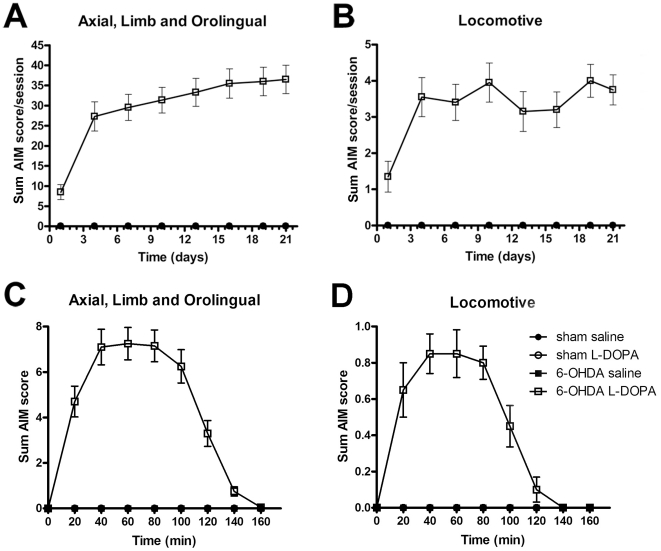
Abnormal involuntary movements induced by chronic treatment with L-DOPA in 6-OHDA-lesioned animals. Time course of changes in dyskinesia evaluated from the sum AIM scores of axial, limb and orolingual (A) or locomotive (B) during L-DOPA (6 mg/kg plus benserazide 12 mg/kg, s.c.) chronic treatment. Time course of sum AIM scores of axial, limb and orolingual (C) or locomotive (D) evaluated after a single injection of L-DOPA the last testing session (21^st^ day); n = 20, 13, 10 and 10 for 6-OHDA L-DOPA, 6-OHDA saline, sham L-DOPA and sham saline, respectively. Note that the sham L-DOPA, sham saline and 6-OHDA saline groups did not develop any abnormal movements.

Chronic L-DOPA administration (6 mg/kg plus benserazide 12 mg/kg) led to a rapid onset of AIMs in the majority of the 6-OHDA-lesioned rats (8/11 in experiment 1). Axial, limb and orolingual AIM scores increased during the first week of treatment and reached a plateau by the second week (Figure 3A). Locomotive AIMs also increased over the time, but showed larger variability between animals and testing sessions (Figure 3B). Sham animals treated with L-DOPA or saline, and 6-OHDA-lesioned animals treated with saline did not develop any abnormal movements.

The time course of the AIMs after a single injection of L-DOPA showed the expected profile (Figure 3C and D). The first signs of dyskinesia were evident 10–20 min after the injection, the peak severity occurred at 40–80 min, and all AIMs subsided by 140–160 min.

### Basal electrophysiological activity in the LC in control and 6-OHDA-lesioned rats following saline or L-DOPA treatment

Three hundred forty-eight noradrenergic neurons were recorded in the LC: 79 neurons from the sham saline group (n = 8 animals), 70 neurons from the sham L-DOPA group (n = 7), 90 neurons from the 6-OHDA saline group (n = 9) and 109 neurons from the 6-OHDA L-DOPA group (n = 12). In the latter group, 77 neurons were recorded from dyskinetic animals (n = 8) and 32 from non-dyskinetic animals (n = 3). Neurons were recorded 24 h after the last injection of saline or L-DOPA. All cells exhibited the typical electrophysiological characteristics of noradrenergic neurons (see “Electrophysiological procedures”) and were localised within the LC.

Neuronal basal firing rate significantly varied between groups (F_(4,346)_ = 12.11, *p*<0.001, one-way ANOVA) (Table 1). Consistent with previous results [28], neurons recorded from the 6-OHDA-lesioned group showed a significantly lower basal firing rate compared to neurons from control animals when treated with saline (p<0.05, Bonferroni’s post-hoc test). After chronic treatment with L-DOPA, neuronal basal firing rate was similar to control values in dyskinetic animals, but non-dyskinetic animals (6-OHDA lesioned treated with L-DOPA) did not differ from 6-OHDA-lesioned animals treated with saline. Significant differences were also found on the coefficient of variation (F_(4,341)_ = 6.95, *p*<0.0001, one-way ANOVA, Table 1). In 6-OHDA lesioned animals treated with L-DOPA, dyskinetic animals had a higher CV value than 6-OHDA lesioned animals treated with saline (p<0.01, Bonferroni post hoc test) but was similar to values obtained in the sham groups. 6-OHDA lesioned animals treated with saline or with L-DOPA (non dyskinetic) showed similar CV values. The latter group had also significantly lower values that those in the sham group (p<0.01, Bonferroni post hoc test). The percentage of neurons with burst-firing activity was significantly reduced by the 6-OHDA lesion (*p*<0.05 vs. sham saline, Fisher’s test, Table 1). Following treatment with L-DOPA, this percentage increased significantly in dyskinetic animals but remained largely reduced in the non-dyskinetic cases, which did not differ significantly from the 6-OHDA saline-treated group. All other electrophysiological properties, including the percentage of spikes occurring in burst, the number of active cells per track and the mean number of spikes per burst, were similar in all groups.

**Table 1:** Control and 6-OHDA lesioned animals.

	sham		6-OHDA lesioned		
	Saline	L-DOPA	Saline	L-DOPA (dyskinetic)	L-DOPA (non-dyskinetic)
**Basal Firing Rate (Hz)**	1.93±0.10 (79)	2.11± 0.10 (70)	1.47±0.07 (90)**	2.10±0.11 (77)&&&	1.31±0.13 (32)###
**Coefficient of variation (%)**	38±1 (79)	37±1 (69)	36±1 (87)	42±1 (76)&&	30±2 (31)##
**% spikes in burst**	2.53±0.50 (38)	2.46±0.51 (37)	3.39±0.73 (28)	3.19±0.84 (45)	5.74±2.32 (5)
**Mean spikes/burst**	2.02±0.03 (38)	2.08±0.05 (37)	2.04±0.04 (28)	2.02±0.02 (45)	2.00±0.00 (3)
**Neurons with burst firing (%)**	48% (38/79)	52% (37/70)	31% (28/90)*	58% (46/77)&&&	16% (5/32)###
**Active cells per track**	4.05±0.48 (8)			3.68±0.23 (4)	

Rats were treated daily with saline or L-DOPA 6 mg/kg in combination with benserazide 12 mg/kg, s.c. for at least 21 days. All neurons were recorded 24 h after the last injection of saline or L-DOPA. Each value represents the mean ± SEM of (n) neurons. Each cell was recorded for 180 seconds. Number of animals included was n = 8, 7, 9, 8 and 3 for sham saline, sham L-DOPA, 6-OHDA saline, 6-OHDA L-DOPA (dyskinetic) and 6-ODA L-DOPA (non-dyskinetic), respectively. The parameter “Active neurons per track” was measured in 10 consecutive tracks of 8 and 4 animals in each group;

* *p*<0.05 and ** *p*<0.01 *vs* sham saline,

## *p*<0.01 and ### *p<*0.001 *vs*. sham L-DOPA,

&& *p<*0.01 and &&& *p<*0.001 *vs*. lesion saline (one-way ANOVA followed by Bonferroni post-hoc test or Fisher’s test for “Neurons with burst-firing”).

### Correlation between the severity of dyskinesia and locus coeruleus basal neuronal activity

**Figure 4:**
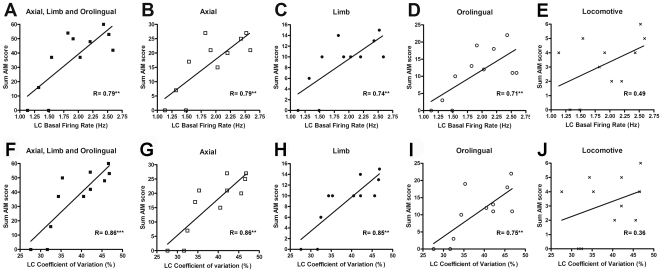
Regression analysis of the severity of dyskinesia and the electrophysiological parameters of locus coeruleus neurons in dyskinetic animals. The electrophysiological parameters of LC neurons are represented on the abscissa and the sum AIMs on the ordinate axis. The basal firing rate was correlated with the sum AIMs (A), and the axial (B), limb (C) and orolingual (D) subtypes. The coefficient of variation also correlated with the sum AIMs (F) and the axial (G), limb (H) and orolingual (I) subtypes. Locomotive behaviour was not correlated with the basal firing rate (E) or with the coefficient of variation in the LC (J). Each symbol represents an animal. Only 6-OHDA lesioned animals treated with L-DOPA (8 dyskinetic and 3 non-dyskinetic) are included in the analysis.

As summarised in Figure 4, regression analyses showed a significant correlation between L-DOPA-induced dyskinesia and both basal firing rate and the coefficient of variation values of LC neurons obtained from each rat. The electrophysiological parameters were obtained 24 h after the last injection of saline or L-DOPA and compared with the AIM score from the last testing session. The sum of axial, limb, and orolingual AIM scores was positively correlated with the analysed parameters. Each subtype of dyskinesia (axial, limb and orolingual), analysed separately, showed significant correlations as well (Figure 4). Identical results were obtained using global AIMs scores (basic scores×amplitude scores) (data not shown). Locomotive AIM did not show any significant correlation with any electrophysiological characteristic of the LC neurons (Figure 4).

### Lack of effect of DSP-4 pretreatment on the development of L-DOPA-induced abnormal involuntary movements

In experiment 2 (cf. Figure 1), 6-OHDA-lesioned animals showing a motor behaviour compatible with a severe unilateral dopamine loss (screened with the cylinder test and amphetamine-induced rotations) received DSP-4 or vehicle injection. DSP-4 would affect retrogradely LC cells by specific degeneration of the noradrenergic nerve terminals. Two weeks later, L-DOPA was administered daily for three weeks until stable dyskinesia scores were achieved.

**Figure 5:**
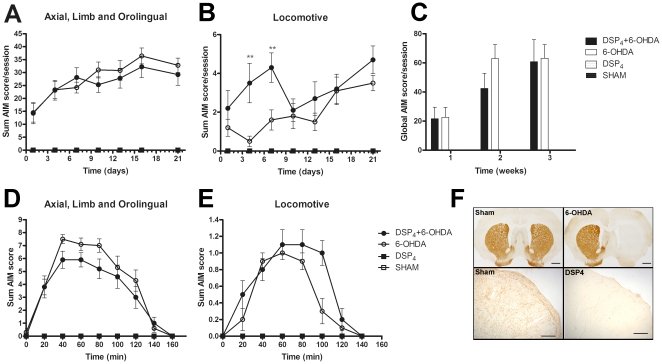
Abnormal movements induced by the chronic treatment with L-DOPA in animals lesioned with 6-OHDA or DSP-4+6-OHDA . The time course of changes in dyskinesia evaluated from the sum (A), locomotive (B) or the global AIM score (C) induced by a 21-day treatment with L-DOPA (6 mg/kg plus benserazide 12 mg/kg, s.c.). Sum and locomotive AIM score was evaluated every testing session and global AIM score weekly. Time course of sum AIM scores of axial, limb and orolingual (D) or locomotive (E) evaluated after a single injection of L-DOPA the last testing session (21^st^ day). Only groups receiving L-DOPA are included in the graph (n = 10, 10, 6 and 5 for DSP_4_+6-OHDA, 6-OHDA, DSP_4_ and sham, respectively). Saline-treated animals, sham L-DOPA and DSP4 groups did not show any sign of dyskinesia. Tyrosine hydroxylase immunostaining in the striatum of sham and 6-OHDA-lesioned animals (F, upper boxes, scale bar 1 mm). Dopamine-β-hydroxylase immunostaining of sham and DSP-4-treated animals in the prefrontal cortex (F, bottom boxes, scale bar 200 µm). **p<0.01 *vs* 6-OHDA (one-way ANOVA followed by Bonferroni post-hoc test).

The chronic administration of L-DOPA induced AIMs exclusively in the DSP4+6-OHDA and 6-OHDA groups. Sham, DSP-4 or saline-injected animals did not show any dyskinetic behaviour. Among the groups that developed AIMs, no differences were observed on either the sum of AIMs or the global AIM scores (*p*>0.05, repeated measures two-way ANOVA) (Figures 5A and C) while the locomotive behaviour showed some group differences (between subjects: F_(3,54)_ = 8.31, *p*<0.001, for group and F_(1,54)_ = 20.71, *p*<0.001, for treatment; within subjects F_(6,324)_ = 5.46 for time, *p*<0.001, repeated measures two-way ANOVA) (Figure 5B). These occurred on the 4^th^ and 7^th^ day of L-DOPA treatment (corresponding to the 2^nd^ and 3^rd^ testing sessions), on which DSP4+6-OHDA lesioned rats showed larger locomotive AIMs than did the group with 6-OHDA lesions only (*p*<0.01 on each day, one-way ANOVA followed by Dunnett’s post-hoc test).

The AIMs time course following a single dose of L-DOPA was similar among the dyskinetic groups (AUC, 588 and 715 for the DSP4+6-OHDA and 6-OHDA-only group, respectively, on the last testing session; *p*>0.05, one-way ANOVA) (Figure 5D). The asymmetric locomotive behaviour tended to be more prolonged in the double-lesion animals (Fig 5E), although the AUC value did not differ significantly between these two groups (AUC, 94 and 68 for the DSP4+6-OHDA and 6-OHDA-only group, respectively, *p*>0.05, one-way ANOVA).

### Impact of locus coeruleus lesioning on L-DOPA-induced abnormal involuntary movements

**Figure 6:**
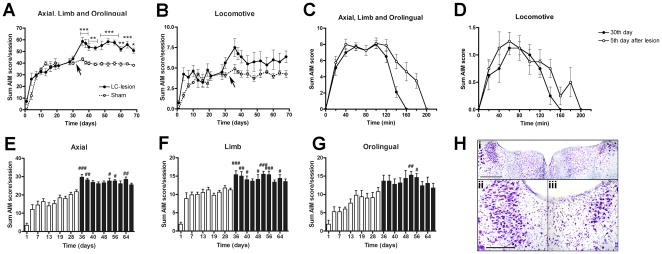
Involvement of the locus coeruleus in the development of abnormal involuntary movements induced by chronic treatment with L-DOPA. Briefly, 6-OHDA-lesioned animals were injected daily with L-DOPA (6 mg/kg plus 12 mg/kg benserazide, s.c.) for 21 days and AIMs were evaluated twice a week. After a 21-day chronic treatment, L-DOPA was administered twice per week. On day 31, animals received an injection of ibotenic acid or vehicle into the right LC (solid line: LC-lesion group, n = 8; dash line: Sham group, n = 5), indicated in the graph with an arrow. Five days after, AIMs were evaluated for another 32 days in 10 additional testing sessions. A significant increase in sum AIM score (A) was observed in the LC-lesion group. Locomotive AIM remained unaltered after the LC lesion (B). In the LC-lesion group, time course of the L-DOPA-induced AIMs after a single injection of L-DOPA was significantly prolonged after the LC damage for the sum (C) but not for locomotive AIM score (D). Bar histograms representing separately axial (E), limb (F) and orolingual AIM scores (G). White or black bars are used for representing each session before or after the LC-lesion, respectively. Thionine-staining in the LC of 6-OHDA-lesioned animals with an additional LC-lesion (Hi). Dashed squares are magnified below, corresponding to the LC contralateral (Hii) and ipsilateral to the lesion (Hiii). All slices are 40 µm-thick and were stained using free-floating slice procedures. Scale bar: 400 µm (Hi) and 200 µm (Hii-Hiii). * *p*<0.05, ** *p*<0.01 and *** *p*<0.001 *vs*. sham group (repeated measures two way ANOVA followed by Bonferroni post-hoc test) # *p*<0.05, ## *p*<0.01 and ### *p*<0.001 *vs*. day 30th (repeated measures ANOVA followed by Dunnett’s post-hoc test) .

To study the impact of a LC lesion in dyskinetic animals, ibotenic acid or vehicle was injected in the LC on the side ipsilateral to the 6-OHDA lesion in L-DOPA-treated rats that had developed stable AIMs (Figure 6; cf. Experimental design in Fig. 1).

Before the LC lesion, the development of dyskinetic movements (axial, limb and orolingual) on the course of the chronic treatment was similar in the LC-lesion and sham groups (F_(1,99)_ = 0.16, *p*>0.05, repeated measures two way ANOVA). Following the LC lesion, the sum of axial, limb and orolingual AIM score was significantly different in the LC-lesion group compared to the sham animals (F_(1,99)_ = 8.31, *p*<0.001, repeated measures two way ANOVA) for all testing sessions (statistical details in the graph) (Figure 6A). The aggravation of dyskinesia remained stable throughout all testing sessions (10 session post-LC lesion during 32 additional days). Identical results were obtained when the global AIMs score was used (data not shown). Asymmetric locomotive behaviour significantly increased during the chronic treatment in a similar way in both groups (F_(1,99)_ = 1.37, *p*>0.05, repeated measures two way ANOVA). No differences were observed after the LC lesion in any group (F_(1,99)_ = 2.34, *p*>0.05, repeated measures two way ANOVA) (Figure 6B).

As illustrated in Figure 6C, in the LC-lesion group the peak-severity of the AIMs following a single administration of L-DOPA was similar before and after the LC lesion. However, the time course of the AIMs was significantly prolonged after the LC lesion. Accordingly, AUC values increased significantly on every testing session after the LC lesion compared to the last day prior to the lesion (day 30^th^ on L-DOPA treatment) (F_(11,77)_ = 9.52, *p*<0.001, repeated measures ANOVA followed by Dunnett’s post-hoc test). Similar results were obtained in the analysis of the global AIM scores (data not shown). On the locomotive scores, no differences were observed after the LC lesion compared with the last day before the lesion (Figure 6D).

In the LC-lesioned group when analysed separately, axial, limb and orolingual AIMs were all significantly aggravated by the LC lesion when compared to the last testing session prior to the LC lesion (F_(19,133)_ = 6.76 for axial, F_(19,133)_ = 24.68 for limb and F_(19,133)_ = 17.98 for orolingual AIMs, *p*<0.001 for all cases, repeated measures ANOVA followed by Dunnett’s post-hoc test) (Figures 6E, F, and G).

### Verification of the lesions

All 6-OHDA-lesioned animals included in the study showed >95% reduction in TH-fibre density in the striatum on the side ipsilateral to the lesion (Figures 5F, upper boxes). DBH-immunostaining revealed a reduction of noradrenergic fibers >79% in the prefrontal cortex in all animals sustaining an intraperitonal injection of DSP-4 (Figures 5F, bottom boxes).

LC lesions produced by local injection of ibotenic acid were verified first by microscopic analysis showing loss of large LC neuron morphology and glial reaction on the lesion side compared to the intact side (Figure 6H). No damage of the surrounding areas was detected. Additional quantification of LC cell bodies by stereological methods showed a mean reduction of 42±4% on the lesion side in all the dyskinetic animals included in the experiment.

## Discussion

The present data constitute the first combined electrophysiological and behavioural study that implicates the LC in L-DOPA-induced dyskinesia. Our results reveal a strong correlation between basic parameters of LC neuronal activity and dyskinesia severity, and moreover show that neuronal damage in this structure increases the L-DOPA-induced AIM scores by prolonging dyskinesia duration.

### Significance of the electrophysiological findings

Similar to the effects observed in some basal ganglia nuclei [51], [52], chronic L-DOPA administration induced changes in the basal electrophysiological properties of LC noradrenergic neurons. We have reproduced our previous results [28] and observed that, in the dopamine-depleted condition, the basal firing rate and the number of neurons with burst-firing in LC cells were significantly decreased. In addition, interesting differences were obtained in the group of 6-OHDA-lesioned animals treated with L-DOPA. LC neurons in dyskinetic animals had a more irregular firing pattern and a higher firing rate, and the number of neurons showing burst-firing activity was larger compared to those that did not developed dyskinesia. Surprisingly, LC neurons in dyskinetic animals displayed electrophysiological characteristics similar to control animals, but 6-OHDA-lesioned animals that did not developed dyskinesia remained similar to 6-OHDA-lesioned rats treated with saline. These data suggest a recovery of the electrophysiological properties towards the control state in dyskinetic rats. The observed difference between dyskinetic and non-dyskinetic animals may depend on a larger brain exposure to L-DOPA and dopamine in the former group. Indeed, LID is associated with increased striatal concentrations of dopamine [42], [52], [53] or L-DOPA [36], [52]. The observed changes cannot be attributed to a direct action of L-DOPA-derived neurotransmitters on adrenergic and dopaminergic receptors because 24 h after the injection, no L-DOPA-derived neurotransmitters are present in the brain [36], [52], [54]. However, it is likely that the daily administration of L-DOPA produces longer term and longer lasting plasticity-type changes that modulate LC neuronal activity. In this sense, a recent publication [55] have reported that 6-OHDA lesion induces an increase of α_2_-adrenoceptor RNA in the LC and that L-DOPA treatment reverses this increment untill control values. It is well known that α_2_-adrenoceptors govern the firing rate in the LC by inhibiting tonically the neuron activity and that raised levels of noradrenaline may downregulate these receptors. Thus, it is bound that our results are directly related to this change in α_2_-adrenoceptor expression induced first by the nigrostriatal degeneration and later by the chronic treatment with L-DOPA.

The correlation between basic parameter of LC neuronal activity and dyskinesia severity is a novel, important contribution of this study. More severe dyskinetic movements had developed in animals in which LC neurons had a higher basal firing frequency and coefficient of variation. The latter parameter, which is a measure of interspike interval regularity, may be indicative of overall changes in neuronal excitability [56]. In addition, an increased release of neurotransmitter has been reported to be associated with burst firing, a form of irregular discharge [57], [58], [59]. Voltamperometric experiments also confirm a very effective transmitter release from the nerve terminal after burst activation [60]. It is possible that in dyskinetic conditions LC neurons increased their activity as part of a homeostatic compensatory response to the prolonged administration of L-DOPA after dopaminergic loss. In fact, drugs that increase noradrenergic activity, mainly α_2_-adrenoceptor antagonists, decrease LID not only in rodents [15], [38], [61], [62] but also in primate models [17], [18], [63] and humans [20]. However, clonidine also reduces dyskinesia. To the best of our knowledge, clonidine is the only α_2_-adrenoceptor agonist reported to improve dyskinesia. Low doses of clonidine decrease noradrenergic cell firing through stimulation of presynaptic α_2_-adrenoceptors [64]. However, at higher doses clonidine also increases LC firing rate through a mechanism involving imidazoline receptors in the LC [65], [66]. It is presently unclear whether α_2_-adrenoceptor agents modulate LID by a direct effect on the LC. Importantly, α_2_-adrenoceptors also are expressed by striatal output neurons and present in other parts of the basal ganglia [67], [68], [69]. Thus, it is possible that treatment with these drugs improves LID not only by acting on the LC but also by their broad modulatory actions on the basal ganglia network.

### Effects of LC lesions on the motor response to L-DOPA

Previous studies investigating the effects of LC lesions on L-DOPA-induced motor complications in 6-OHDA-lesioned rats have produced some contradictory results. For example, additional noradrenergic denervation with DSP-4 although selectively increased number of animals that respond to L-DOPA, was reported not to modify circling behaviour [31], wearing-off type fluctuations [32], or L-DOPA-induced dyskinesia [70]. In other studies, the depletion of LC noradrenaline with either DSP-4 or local 6-OHDA lesions produced a potentiation of parkinsonian-like symptoms [71], [72], an increment of apomorphine-induced circling behaviour [73], and anticipated the onset and worsened the severity of L-DOPA-induced AIMs [30]. In the present study, we used two approaches to investigate the role of LC in dyskinesia. First, DSP-4 was used to induce the retrograde degeneration of LC noradrenergic projections. In this experiment, dyskinetic movements that developed in double DSP4/6-OHDA-lesioned rats were in all respect similar to those seen in 6-OHDA-only animals. Second, ibotenic acid was used to produce an acute and complete lesion of the LC in dyskinetic animals. In this case, the LC was destroyed once dyskinesia was already established, and the AIM scores increased significantly after the lesion. DSP-4 is a neurotoxin highly specific for the nerve terminals originating from the LC [74], [75] whose projections retrogradely degenerate [76]. However, after DSP-4 administration, a slow and gradual recovery of noradrenaline stores back to control values has been reported [77], [78]. From the electrophysiological point of view, noradrenergic depletion with DSP-4 leads to transient electrophysiological changes in noradrenergic pathways [79], [80]. Moreover, mice whose central noradrenaline levels have been depleted by prior treatment with DSP-4, the remaining noradrenergic neurons respond with an increase in noradrenaline turnover after L-DOPA loading [81]. In the present study, the ascending noradrenergic fibres were notably affected by the DSP-4 injection. LC-cell bodies quantification was not performed, however, a recent paper performed partially in our laboratory, has reported that despite decreasing cortical noradrenaline levels and affect noradrenergic neuron terminals, DSP-4 does not affect the functioning of LC neurons or selectively lesion LC noradrenergic cell bodies [34]. The ability of LC cells to survive the damage from DSP-4 and the questionable selectivity of the neurotoxin for LC innervation may be relevant for the understanding of our results. These negative results are countered by the effects of the ibotenic acid lesion, resulting in a noticeable increase in dyskinesia duration. These results are consistent with another study that showed an aggravation of dyskinetic movements in 6-OHDA-lesioned animals when the damage to the noradrenergic innervation was enhanced by omitting desipramine pretreatment prior to the MFB lesion surgery [30]. Altogether, these results indicate that the role of the LC in LID may become evident only when an important degree of degeneration has occurred.

Asymmetric locomotive behaviour was the only abnormal motor response that did not correlate with LC electrophysiological properties and did not follow the same increase as the other abnormal movements after LC destruction. In fact, this behaviour is not considered a valid measure of dyskinesia because it is induced by drugs with very low dyskinesiogenic potential, and because it is not attenuated by compounds that have anti-dyskinetic efficacy in patients [15], [38].

### Possible mechanisms by which the LC modulates L-DOPA-induced dyskinesia

The most recent studies on mechanisms of L-DOPA-induced dyskinesia have crystallized three main layers of alterations, namely: (1) presynaptic dysregulation of dopamine release and clearance; (2) maladaptive molecular and synaptic plasticity in striatal neurons; (3) changes in firing patterns in the basal ganglia output nuclei, including the internal segment of the globus pallidus (GPi), and the substantia nigra pars reticulata (SNpr) (recently reviewed in [82]). Because of their widespread distribution, adrenoceptors and noradrenergic projections may potentially modulate each of the above processes, and the following discussion will focus only on mechanisms that are supported by sufficient information. At the presynaptic level, the noradrenergic system plays an important role in the clearance of L-DOPA-derived dopamine in 6-OHDA-lesioned rats [54]. Indeed, noradrenergic fibres can contribute both to the enzymatic breakdown of dopamine via monoamine oxidase-A (MAO-A) [83], [84] and to dopamine reuptake via the noradrenaline transporter [54]. When both the noradrenergic and dopaminergic systems are damaged, L-DOPA-derived dopamine is likely to remain for a longer time in the brain extracellular space, an effect that would lead to a prolongation of dyskinesia. A presynaptic mechanism may therefore explain an increased duration of L-DOPA-induced AIMs following LC lesion, as observed in the present study. Other important effects of the noradrenaline system may occur at the level of the basal ganglia output nuclei. In the rat, the expression of L-DOPA-induced dyskinesia is associated with distinct neurochemical and electrophysiological changes in the SNpr, consisting in large elevations of extracellular dopamine and GABA levels [42], [85] accompanied by altered neuronal firing patterns [52]. The α_2_-adrenoceptors modulate GABA release in the SNpr [86], and α_1_- activation increases the excitability and firing rate in SNpr neurons [87]. Although low, a noradrenergic reuptake site has also been observed in the SNpr [88], [89]. Interestingly, a recent publication has revealed that the LC lesion increases the basal firing rate in the SNpr in 6-OHDA-lesioned rats [73].

### Conclusions

The study of the impact of non-dopaminergic systems on the development of dyskinesia will bring new light to the understanding of PD. In this work, we have demonstrated that the LC noradrenergic system is involved in the pathophysiology of LID from both an electrophysiological and a behavioural standpoint. The modulation of this system may constitute an important therapeutic approach to the treatment of PD and of the complications associated with L-DOPA pharmacotherapy.
